# A matching pursuit algorithm for inferring tonic sympathetic arousal from spontaneous skin conductance fluctuations

**DOI:** 10.1111/psyp.12434

**Published:** 2015-04-30

**Authors:** Dominik R. Bach, Matthias Staib

**Affiliations:** ^1^ Department of PsychiatryPsychotherapyand PsychosomaticsUniversity of ZurichZurichSwitzerland; ^2^ Wellcome Trust Centre for NeuroimagingUniversity College LondonLondonEngland

**Keywords:** Electrodermal, Anxiety, Normal volunteers

## Abstract

Tonic sympathetic arousal is often inferred from spontaneous fluctuations in skin conductance, and this relies on assumptions about the shape of these fluctuations and how they are generated. We have previously furnished a psychophysiological model for this relation, and an efficient and reliable inversion method to estimate tonic arousal from given data in the framework of dynamic causal modeling (DCM). Here, we provide a fast alternative inversion method in the form of a matching pursuit (MP) algorithm. Analyzing simulated data, this algorithm approximates the true underlying arousal up to about 10 spontaneous fluctuations per minute of data. For empirical data, we assess predictive validity as the ability to differentiate two known psychological arousal states. Predictive validity is comparable between the methods for three datasets, and also comparable to visual peak scoring. Computation time of the MP algorithm is 2–3 orders of magnitude faster for the MP than the DCM algorithm. In summary, the new MP algorithm provides a fast and reliable alternative to DCM inversion for SF data, in particular when the expected number of fluctuations is lower than 10 per minute, as in typical experimental situations.

Spontaneous fluctuations (SF) in skin conductance (also termed nonspecific electrodermal responses) are commonly used to infer a central state of tonic sympathetic arousal (tSA) (Boucsein, 2012), for example, due to cognitive load, stress, or anxiety (Bach & Erdmann, 2007; Bach, Erdmann, Schmidtmann, & Monnikes, 2006; Erdmann & Baumann, 1996). The number of SF per time unit is among the most widely used indices of tSA (Boucsein, 2012) and is a better predictor of anxiety than the amplitude of SF (Bach, Friston, & Dolan, 2010). However, the identification of SF from skin conductance recordings is nontrivial and requires assumptions about their shape, often embodied in the expertise of researchers performing visual scoring (Boucsein, 2012). At the same time, beyond amplitude thresholds there is no clear community consensus on formalized assumptions or analysis algorithms (Boucsein et al., 2012).

We have previously furnished a formal approach to fully automated analysis that removes subjective and potentially biasing elements embedded in visual inspection or semiautomated analysis of SF. This approach is embodied in a psychophysiological model (PsPM) of how tSA causes SF: tSA→SF. This model can be inverted probabilistically to directly infer the most likely tSA, given skin conductance measurements (Bach, Daunizeau, Kuelzow, Friston, & Dolan, 2011). Similar approaches have been proposed for inferring phasic SA (Bach, Daunizeau, Friston, & Dolan, 2010; Bach et al., 2011; Bach, Flandin, Friston, & Dolan, 2009; Bach & Friston, 2013; Bach, Friston, & Dolan, 2013).

Our PsPM splits up the relation tSA→SF into two relations: tSA→SN→SF, where SN stands for the sudomotor nerve activity that causes SF. A neural model tSA→SN defines how SA generates SN activity. Physiological investigation has demonstrated that SF occur in the absence of external events, and are preceded by compact firing bursts of SN activity, innervating the respective skin region (Macefield & Wallin, 1996; Ogawa & Sugenoya, 1993; Sugenoya, Iwase, Mano, & Ogawa, 1990). The neural model therefore defines compact short SN bursts, the number of which is a linear function of tSA. The peripheral model SN→SF is a biophysical model that specifies how SN activity generates SF in the form SF = SN*SCRF, where * is the convolution operator, and SCRF is a “canonical” skin conductance response function.

Because the onset of SN is not specified in this PsPM, linear methods are difficult to apply for model inversion. Consequently, the PsPM is formulated in terms of nonlinear dynamic equations, and inverted by a variational Bayes algorithm developed in the framework of dynamic causal modeling (DCM; Daunizeau, Friston, & Kiebel, 2009). While this method produces reliable and sensitive estimates of tSA, the inversion requires evaluation of high‐dimensional multivariate probability distributions and is necessarily slow. Here, we sought to develop and evaluate a fast inversion routine that closely approximates the tSA estimates derived by DCM inversion.

Matching pursuit (MP) is a machine‐learning algorithm that seeks to decompose data into a small set of functions (termed *atoms*), defined in an overcomplete set of possible functions (termed *dictionary*; Mallat & Zhang, 1993). In our case, the dictionary consists of all individual SF that could possibly be generated under the previously defined PsPM. The dictionary is iteratively searched by a time‐efficient, heuristic algorithm. On each iteration, the algorithm identifies one atom that on its own explains the maximum variance in the data. The contribution of this atom is then subtracted from the data, and the next iteration starts. The algorithm only considers one atom at a time and selects the best individual atom. This means it can possibly miss a combination of atoms that together better explain the data but in which the individual atoms explain less variance. Algorithms combining locally optimal solutions rather than optimizing the final solution are termed *greedy* in the machine learning literature. Such algorithms are suboptimal by design but often simpler and/or faster than other classes of algorithms.

In order to count the number of SF, our previous DCM uses an amplitude criterion as generally recommended (Boucsein et al., 2012). Hence, a precise amplitude estimate is required for each SF. To achieve this, we complemented the MP algorithm by subsequently reestimating the contribution of all identified atoms simultaneously. In summary, we hypothesized that MP could provide an approximation to the set of SF that most likely constitutes the measured data and thereby achieve the same goal as the original DCM algorithm, presumably with less precision but much faster.

Hence, in the present paper, we sought to investigate the inversion results and the computation time required by these two algorithms. First, we report the precision with which both algorithms detect the known structure of simulated data. Second, we analyze three datasets of skin conductance recordings during public speaking anticipation and mental load to examine the sensitivity of the algorithms to infer tSA, that is, their predictive validity.

## Method

### Simulated Data

Simulated data were required to fulfill three criteria: (1) They should be generated under the same PsPM used for data analysis in order to benchmark the algorithms under noiseless conditions, (2) the number of SF per data segment should reflect a certain range such as to evaluate the algorithm in different situations, and (3) simulated SN firing should occur in separable, compact bursts to reflect physiological SN firing. Burst separability is not required in the PsPM and is therefore an additional criterion.

We simulated 30,000 SN traces of 60‐s duration and 10 Hz sampling frequency, containing between 1 and 30 spontaneous SN bursts, modeled as Gaussian bumps with 0.3 s standard deviation. SN traces were then entered into the peripheral ordinary differential equation model described in Bach et al., 2011, to generate synthetic skin conductance data. Amplitude of the SN bursts was randomly drawn from a uniform distribution between 0.1 and 2.0 units, where an SN burst with unit amplitude causes an SF with 1 µS amplitude.

Onset of the SN bursts was iteratively generated from a Poisson process; that is, their occurrence was uncorrelated, with the restriction that bursts were separated by at least 1 s. Burst frequencies ranged from 1 to 30 per minute. Starting with the lowest burst frequency, we used the reciprocal of each desired burst frequency as mean parameter for an exponential distribution from which we randomly drew interburst intervals and added 1 s. Resulting burst onset sequences were then binned according to the number of SN bursts they contained. We used the first 1,000 sequences generated for each desired number of SN bursts.

### Participants

We reanalyzed two public speaking datasets from the same laboratory, both of which are based upon a similar paradigm and were used to develop the DCM method (Bach et al., 2011). A third (as yet unpublished) dataset was obtained to explore the predictive validity of the algorithms to infer tSA under mental load. Dataset 1 served as training dataset, which we used to optimize the amplitude threshold for counting SF. Datasets 2 and 3 served as independent validation datasets.

Dataset 1 contained four measurements from each of 40 healthy male university students (18–35 years) who participated in a public speaking anticipation paradigm with a repeated measures factorial design (Bach & Erdmann, 2007). Focus of the study was the interaction of habitual and situational symptom focusing, operationalized as attention toward neck muscle tension. The main experimental manipulation had no effect on indices of skin conductance, and data from the different experimental groups were combined for the present analysis, where we focus on the effect of the public speaking treatment. There were two baseline measurements, one measurement after the announcement of a public speech, and another after disclosure of the speech topic. This manipulation was originally carried out in order to separate effects of anxiety and cognitive load.

Dataset 2 included four measurements for each of 32 healthy female university students (19–29 years) who underwent a similar public speaking experiment in a between‐subjects design. That is to say, half of the participants were to deliver a public speech, and the other half a speech without an audience. There was one baseline measurement, one measurement after announcement of the speech, one after disclosure of the topic, and one immediately before the speech. Fourteen of 128 epochs contained motion artifacts and were excluded, which removed one participant from analysis altogether for whom the baseline period could not be used.

In Dataset 3, 20 healthy participants (18–28 years, 11 female) were assigned on a between‐subject level to either an arithmetic or an attention task. Each subject underwent both a resting and a treatment condition. In the arithmetic task, participants were tasked to mentally add three seven‐digit numbers that were displayed on a computer screen. They were informed that the experimenter would provide them with pen and paper 2 min after display onset to write down the result. In the corresponding attention task, the same three numbers were presented for 2 min with the instruction to attend them. Additionally, each participant was instructed to relax for another 2 min in front of a blank computer screen. Order of the resting and treatment epochs was balanced across participants within the two conditions.

### SCR Recordings and Preprocessing

After skin cleansing with propanol (Dataset 1, 2 only) and a resting period of 30 min to allow for electrolyte equilibrium, skin conductance was recorded on thenar/hypothenar of the nondominant hand using 8 mm Ag/AgCl cup electrodes (Coulbourn, Whitehall, PA) and 0.5% NaCl electrode gel (Par, Berlin, Germany); 0.5 V constant voltage was provided by a S77‐21 coupler (Coulbourn). The signal was band‐pass filtered (Dataset 1, 2 only, 0.0159 and 5 Hz), digitally converted with 10 Hz (Dataset 1), 100 Hz (Dataset 2), or 1000 Hz (Dataset 3) sampling rate (DI‐205, Dataq, Akron, OH), and recorded (Windaq, Dataq). For Dataset 3, data were digitally band‐pass filtered (unidirectional Butterworth filter, 0.0159 and 5 Hz). All data were downsampled to 10 Hz resolution before analysis.

### MP Inversion

#### Creation of a dictionary

The overcomplete dictionary specifies all SF that could possibly be observed under the forward model. In order to specify these, we defined SN bursts as Gaussian bumps with unit amplitude, 0.3 s standard deviation, centered on time points ranging from −9 s relative to data onset to +1 s relative to data offset in steps of 0.1 s, corresponding to the time resolution of the data. These SN bursts were then used as input to the peripheral model, embedded in an ordinary differential equation as specified previously (see Appendix of Bach, Daunizeau, Kuelzow, Friston, & Dolan, 2011). Note that this differential equation does not take into account conduction delay. Conduction delay is subtracted from SN burst latency estimates if the time point of central SF generation is required.

#### Greedy search algorithm

The matching pursuit algorithm finds on each iteration the atom (*g*) from dictionary (*D*), which best explains the residual signal (*R*_n_), by maximizing the similarity between the signal and the chosen atom. This similarity is quantified as signed inner product and reflects the amplitude of an SN. The contribution of this atom to the residual signal is then subtracted, and the new residual signal analyzed to find the next atom. In pseudocode, the algorithm is:

$R_{1}=data$; *n* = 1

Repeat

find $g_{\gamma_{n}}\in D$ that maximizes the inner product $<g_{\gamma_{n}},R_{n}>$
$a_{n}=<g_{\gamma_{n}},R_{n_{}}>$
$R_{n+1}=R_{n}-a_{n}g_{\gamma_{n}}$
n = n + 1

until ${\left\|R_{n}\right\|}_{2}<ɛ$ or *n* > max*n*, or $a_{n}\leq 0$

with ɛ = (0.001N)^1/2^ and max*n* = 30 per minute of data N: number of data points

In the original formulation of the MP algorithm, the absolute inner product is maximized on each iteration (Mallat & Zhang, 1993)—this is maximizing the explained variance. However, negative weight values imply negative SF, which are biophysically impossible. Therefore, only positive weights were accepted, which is achieved by maximizing the signed inner product. Finally, the indices of the matched dictionary atoms define the onset of the estimated SF.

Our previous DCM modeled a fixed number of 30 SF per minute of data. This is why we chose a maximum number of 30 SF of data as a stopping criterion for the algorithm. Alternatively, if the residual sum of squares was below a threshold ε, or new atoms with positive contribution could be identified, the algorithm would stop.

#### Reestimation of SF amplitude

When two SF overlap, the greedy algorithm will overestimate the amplitude of one and underestimate the amplitude of the other, and this phenomenon can impede scoring of above‐threshold SF. Hence, in a final step, the amplitudes (*a*) of all identified atoms (*g*) are reestimated by using them as predictors in a multiple regression model and estimating their respective weights. These weights then serve as amplitude estimates of the SN bursts causing each SF. The number of above‐threshold bursts is taken as the estimate of tSA. This reestimation is meant to improve scoring of above‐threshold SF, not for precise amplitude estimation of above‐threshold SF.

The algorithm is freely available as function scr_sf_mp in the software package PsPM (which includes the package SCRalyze) and can be downloaded from http://pspm.sourceforge.net.

### DCM Algorithm

For benchmarking, we inverted all simulated and experimental data with a previously published DCM algorithm (Bach et al., 2011). This algorithm finds the SN amplitude and onset parameters that best explain the data, by considering all parameters simultaneously using a variational Bayes approach (Daunizeau, Friston, & Kiebel, 2009). We modeled 30 SF per minute of data, analogous to the MP settings and previous work. Inversion was performed using version b2.1.8 of the package SCRalyze (http://pspm.sourceforge.net).

### Visual Scoring

For comparison, all datasets were visually scored by a trained expert. Files were automatically renamed with random file names, such that the scorer was blind to the treatment condition. We used the MS‐DOS—based software Event Detection and Analysis (E.D.A.; Kayser & Trosiener, 1993; Trosiener & Kayser, 1993) within a Windows PowerShell. This program prepares analysis by providing automated peak detection using the tool EventDetection; visual scoring is done in a graphic interface using the tool EventCheck. Initial threshold was 0.01 µS. Note that in a previous publication comparing DCM and visual scoring of Dataset 1 and 2, visual analysis was done in the context of the original investigations and by different experts. To ensure comparability across all three datasets, analyses for the present work were performed by the same expert (MS).

### Timing Considerations

To assess the computation time required for each inversion, each dataset was sequentially inverted by DCM and by MP, one immediately after the other. All computations were performed on one core of a Dell Precision T3600 workstation with a 4‐core CPU (Intel Xeon E5‐1620), a clock rate of 3.6 GHz, and 16 GB RAM, operated under Windows 7 Professional and MATLAB R2012b.

### Simulation Benchmarking

In simulated data, ground truth is known. We report the estimated number of SF as a function of the true number of SF, and the root mean squared error (RMSE) between true and estimated number of SF. For benchmarking of timing and amplitude estimates, we matched each estimated SF to the true SF that was closest in time, starting with the pair of true/estimated SF with the smallest time difference. Each true and each estimated SF was considered only once. In case of mismatch in the number of true/estimated SF, this procedure was continued until no pair could be formed any more. We then computed the RMSE of amplitude and timing estimate for these pairs, ignoring unmatched true/estimated responses, across all simulations.

### Empirical Data Benchmarking

Sensitivity of SCR analysis methods to recover a known ground truth has been cast as a model comparison (Bach, Daunizeau, Friston, & Dolan, 2010; Bach et al., 2013), as a classification problem (Bach, Friston, & Dolan, 2010), or as a search for the highest test statistics for a given contrast (Bach et al., 2009; Barry, 1990). These approaches are all equivalent in determining the most sensitive method, but the model comparison approach also allows a principled statement of whether a method is significantly more or less sensitive than another method. Hence, we report sensitivity in terms of a log Bayes factor (LBF)—the difference in log model evidence between the MP results and the DCM results as reference. According to the definitions used here, lower LBF indicates higher model evidence (i.e., higher sensitivity to distinguish the two experimental conditions).

Specifically, we used a general linear model with the contrast of interest as the response variable, and the estimated SA as predictor. Contrast of interest were (a) public speaking anticipation versus rest for Dataset 1, (b) public speaking anticipation versus private speaking anticipation for Dataset 2, (c) mental load versus rest for Dataset 3, and (d) mental arithmetic versus attention for Dataset 3. For between‐subject contrasts 2 and 4, estimated tSA was calculated per participant as tSA(treatment) – tSA(rest). For contrasts 1 and 3, the design matrix included subject effects and the response estimates per epoch. For contrast 2, tSA (treatment) was computed as average tSA in the three public speaking conditions. For all contrast, the design matrix additionally included an intercept. This is equivalent to an independent samples *t* test (contrasts 2/4) and a paired *t* test (contrasts 1/3). In both cases, this approach tests whether tSA estimates for the two different states are drawn from distributions with different means. This approach allows computing a residual sum of squares (*RSS*), which was converted to a negative log likelihood value (*NLL*), such that smaller NLL values indicate a higher predictive validity using the following relation taken from Burnham and Anderson, 2004:

$$NLL=n\;log\left(\frac{1}{n}RSS\right)$$

where *n* is the number of observations. This disregards model complexity, which was the same for all analyses of a particular dataset. LBF is the difference in *NLL* between a given method and the reference method. Here, DCM with the currently recommended amplitude threshold of 0.1 µS was used as reference method. An absolute LBF of >3 is often regarded as decisive, by analogy to a classic *p* value. If a classic test statistic falls into the rejection region, the probability of the data given the null hypothesis is *p* < .05. For an LBF > 3, the probability of the null hypothesis given the data is 1/exp(3) ≤ .05 (Penny, Stephan, Mechelli, & Friston, 2004; Raftery, 1995).

We also computed paired *t* tests for the winning amplitude threshold, to facilitate an intuitive understanding of the difference between the methods. LBF and *t* value are monotonically related—higher *t* values translate to lower LBF and indicate higher sensitivity.

## Results

### Simulations

**Figure 1:**
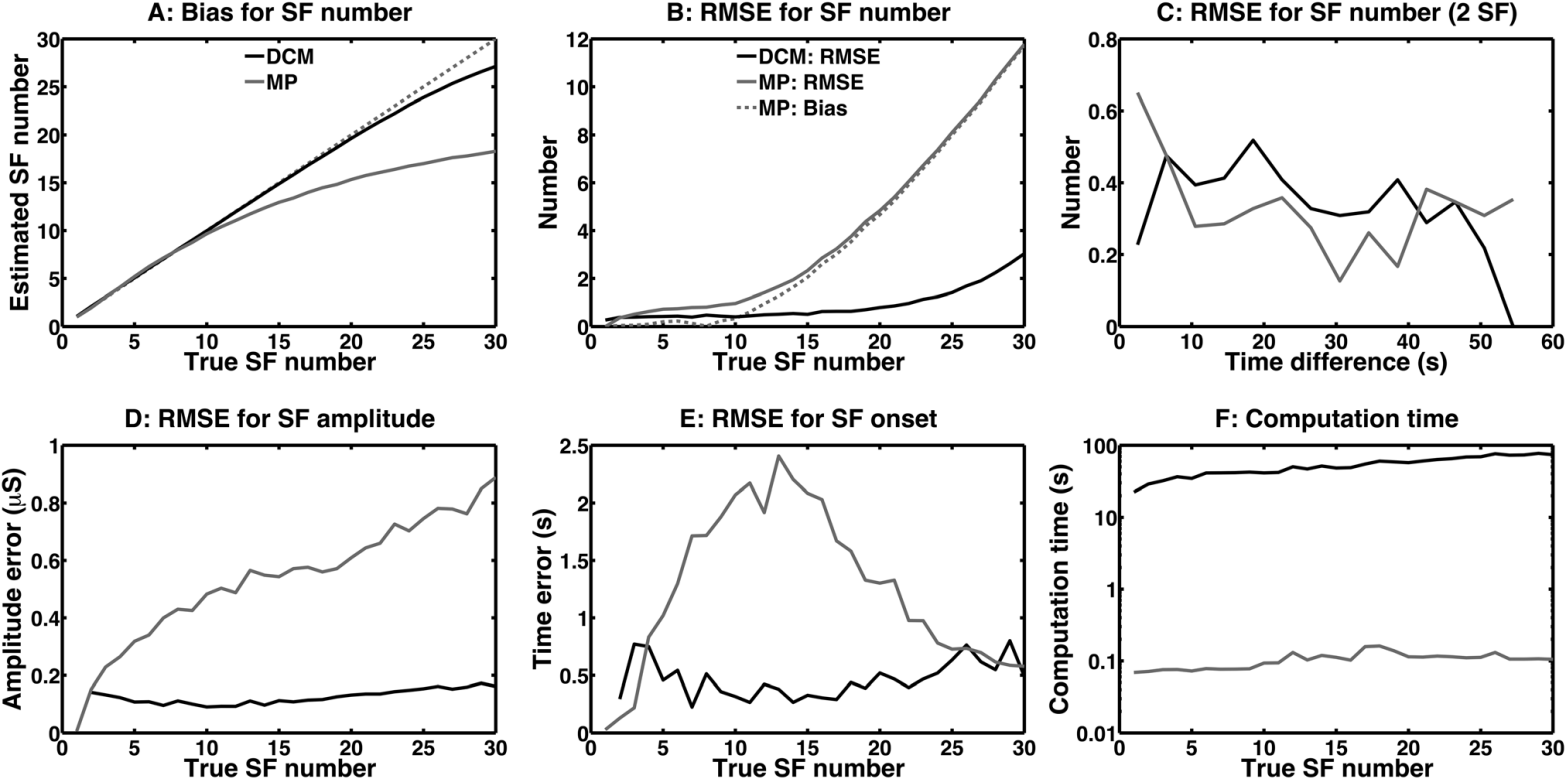
Estimation of SF from simulated skin conductance data. A: True and estimated SF number. Dotted line: perfect correspondence between true and estimated SF number. B: root mean squared error (RMSE) of the estimated SF number, in dependence on true number of SF. Dotted line: RMSE predicted by a bias in SF number estimation using MP. C: RMSE of estimated SF number for 2 true SF, in dependence on their separation in time. D: RMSE of SF amplitude estimates. E: RMSE of SF onset estimates. F: Computation time per minute of data (logarithmic scale).

Inversion results for different true SF numbers per minute are shown in Figure 1 The DCM inversion yields unbiased SF number estimates for up to 20 SF, while MP starts to underestimate the true SF number for 10 SF and more per minute. This bias almost entirely explains the higher RMSE in the MP estimation of SF number (Figure 1B).

We then analyzed how the separation in time of true SF affects their estimation. For 2 SF per minute, the RMSE of the MP algorithm is particularly high when the true SF are separated by less than 4 s, while DCM is not particularly affected by the overlap of true SF. On the other hand, RMSE of the DCM algorithm is particularly low when they are separated by more than 50 s, while MP does not benefit from this separation in time.

SF amplitude and onset are not commonly analyzed but might be of interest in special applications (Boucsein et al., 2012). The RMSE of the DCM algorithm in terms of estimating amplitude and onset does not depend on SF number. The RMSE of the MP algorithm, however, increases linearly with SF number both for amplitude and for onset estimation. For onset estimation, the maximum RMSE is reached at about 15 SF per minute. Beyond that, RMSE decreases—this is due to the fact that each estimated SF will be matched with the closest true SF for analysis. As the number of SF increases, the closest true SF will, on average, be closer in time.

While the MP algorithm performs generally worse in terms of precision, it is also much faster. DCM computation time was in the range of 10–100 s per minute of data and linearly increased with the number of true SF (while the number of modeled SF was always set to 30). In contrast, MP computation time was below 0.1 s per minute of data and hence 2–3 orders of magnitude smaller.

### Empirical Data

**Figure 2:**
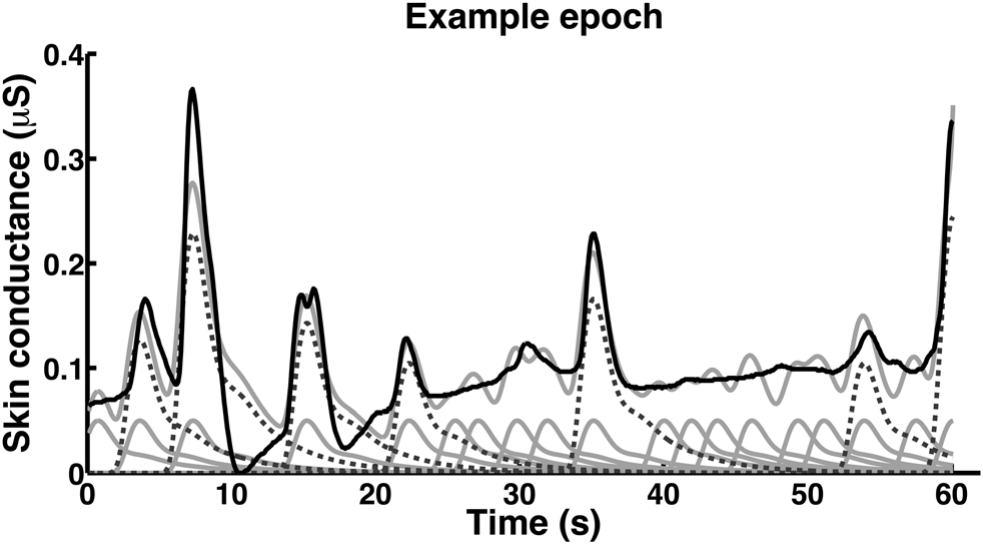
MP inversion of an example epoch from Dataset 1. Black line: filtered skin conductance data. Light gray lines: data fit after MP inversion, and individual atoms of the solution, shown with standardized amplitude. Dotted dark gray lines: individual atoms with above‐threshold amplitude of the final solution, shown with the reestimated amplitude.

Figure 2 shows how the MP algorithm inverts an example epoch. Inversion was terminated in most cases because no further atoms with positive contribution could be identified (90, 105, and 30 cases for the three datasets, respectively), and in the remaining cases because the squared error criterion was fulfilled (70, 55, and 10 cases for the three datasets, respectively). The algorithm never reached the maximum allowed number of SF.

**Figure 3:**
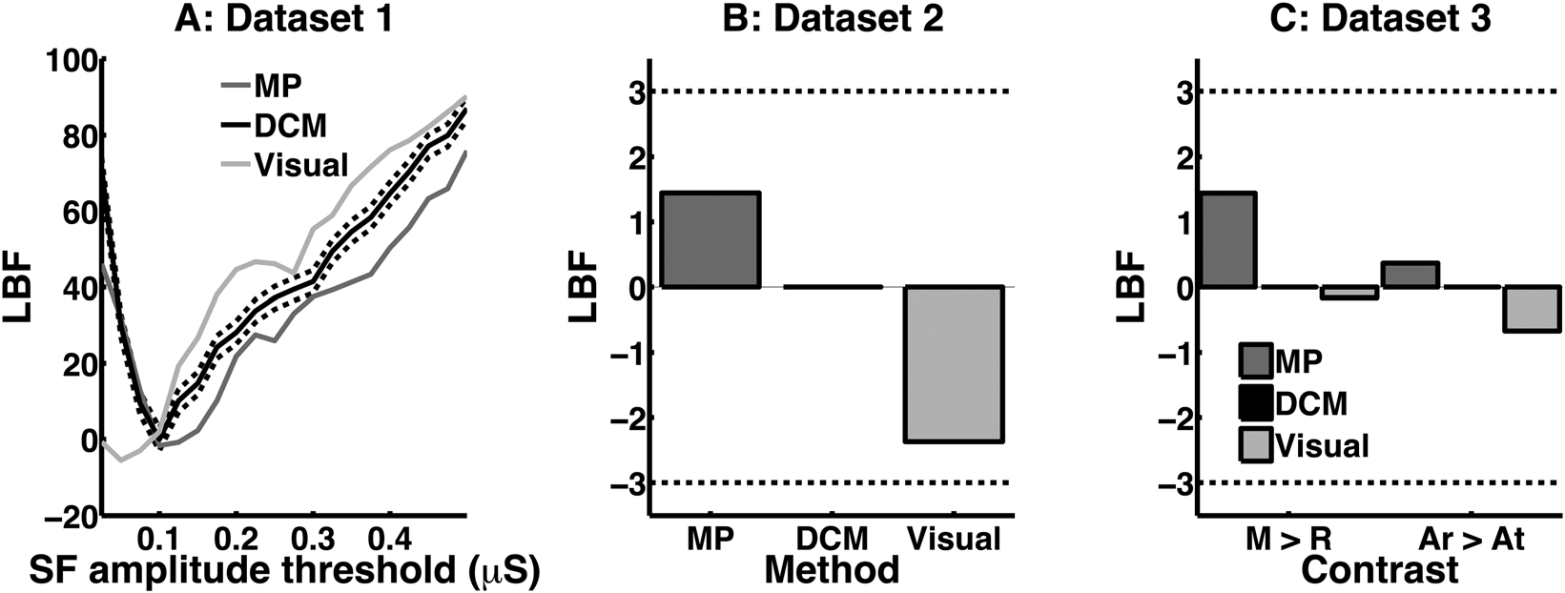
Predictive validity of the three algorithms, expressed as log Bayes factors (LBF) with respect to the reference method (DCM with amplitude threshold of 0.1 µS). LBF is lower when the estimated tSA better separates two known psychological states (Dataset 1: rest vs. public speaking, Dataset 2: public vs. nonpublic speaking, Dataset 3: mental load vs. rest). An absolute LBF of 3 is often considered significant and is indicated by a dotted light gray line. A: Predictive validity for DCM, MP and visual scoring estimates in dependence on amplitude threshold in Dataset 1. B: Predictive validity for Dataset 2, using the optimal amplitude threshold for each algorithm as derived from Dataset 1. C: Predictive validity for Dataset 3, using the optimal amplitude threshold for each algorithm as derived from Dataset 1. M > R: mental load vs. rest (within‐subject comparison). Ar > At: arithmetic vs. attention (between‐subjects comparison of within‐subjects differences, corresponding to an interaction).

Following model inversion with DCM and MP, we analyzed how amplitude thresholds for counting SF affect predictive validity in Dataset 1. Figure 3A shows that both algorithms achieved maximum predictive validity in Dataset 1 at a threshold of 0.1 µS. The two methods were not significantly different at this threshold, as indicated by an absolute LBF difference smaller than 3. The *t* statistic for comparing public speaking anticipation versus rest was, for the DCM estimates, *t*(39) = 9.0, and for the MP estimates, *t*(39) = 9.6. Table 1 shows the tSA estimates obtained by the different methods. Computation time was much faster for MP than for DCM (Table 2).

**Table 1:** Means (Standard Deviations) Across Subjects of the Estimated Number of SF Per Minute of Data, for the Different Algorithms

	DCM		MP		Visual scoring	
	Rest	Treatment	Rest	Treatment	Rest	Treatment
Dataset 1						
Public speaking anticipation	3.1 (3.1)	8.2 (3.7)	3.8 (2.9)	8.5 (3.5)	3.9 (3.5)	8.6 (4.2)
Dataset 2						
Nonpublic speaking anticipation	3.7 (5.1)	3.5 (3.2)	4.3 (5.1)	4.5 (3.0)	2.9 (3.4)	2.5 (2.5)
Public speaking anticipation	4.1 (4.5)	8.4 (3.7)	4.6 (4.3)	8.6 (3.2)	3.4 (4.0)	6.7 (3.3)
Dataset 3						
Attention	7.1 (8.2)	10.5 (10.7)	7.8 (6.6)	9.7 (6.7)	3.8 (3.9)	5.2 (4.3)
Arithmetic	4.2 (3.0)	11.3 (8.5)	7.1 (4.0)	10.9 (6.4)	4.1 (2.3)	7.8 (5.0)

**Table 2:** Computation Time in Seconds per Minute of Data, for the Two Algorithms Under Study

	DCM	MP
	*Mean* ± *SD*	*Mean ± SD*
Dataset 1	40.2 ± 21.5	0.12 ± 0.08
Dataset 2	44.7 ± 63.7	0.10 ± 0.02
Dataset 3	77.5 ± 34.4	0.15 ± 0.04

*Note*. In contrast, visual scoring took approximately 72 s per minute of data, across all datasets. Computation time for the preparatory automated peak detection was not analyzed.

This amplitude threshold was then used for Datasets 2 and 3. Again, predictive validity was not significantly different between the two methods for both datasets (Figure 3B,C). The *t* statistic for comparing the two conditions in Dataset 2 was, for the DCM estimates, *t*(29) = 3.1, and for the MP estimates, *t*(29) = 2.8. For distinguishing mental load from rest in Dataset 3, *t* statistics were *t*(19) = 2.7 for DCM estimates, and *t*(19) = 2.0 for MP estimates. To differentiate mental arithmetic from attention, *t* statistics were *t*(18) = 0.9 for DCM estimates and *t*(18) = 0.7 for MP estimates.

In an exploratory approach, we also extracted the optimal amplitude threshold for Datasets 2 and 3. In Dataset 2, best predictive validity was achieved for DCM with a threshold of 0.225 µS (LBF = −5.1), and for MP with a threshold of 0.175 µS (LBF = −6.5). Both were thus significantly better than the reference method (DCM with a threshold of 0.1 µS), but with no significant difference between MP and DCM at the optimal threshold. In Dataset 3, best predictive validity was achieved for DCM with a threshold of 0.1 µS for both contrasts. For MP, the best threshold was at 0.15 µS (LBF = −0.8, mental load vs. rest) and 0.125 µS, respectively, (LBF = −0.2, arithmetic vs. attention). Hence, at the optimal thresholds, MP was not significantly better than at the predetermined threshold, or than the reference method.

### Comparison with Visual Scoring

As a comparison, a trained expert visually scored all SF, using an initial threshold of 0.01 µS. For Dataset 1, best predictive validity was achieved at a threshold of 0.05 µS and was, with an LBF = −5.5 at this threshold, significantly better than the reference method (DCM with a threshold of 0.1 µS). Using this amplitude threshold for Datasets 2 and 3, predictive validity of visual scoring was not significantly different from the reference method. Best predictive validity for these datasets was achieved with a threshold of 0.075 µS (Dataset 2, contrast arithmetic vs. attention for Dataset 3) and 0.05 µS (contrast mental load vs. rest for Dataset 3). For Dataset 2, visual scoring at the optimal threshold had significantly lower predictive validity than DCM at the optimal threshold (0.225 µS, LBF difference: −4.1 in favor of DCM). For Dataset 3, DCM and visual scoring were not significantly different at the optimal threshold.

## Discussion

Inferring tSA from the number of SF in skin conductance data requires assumptions about their shape, which we have previously embodied in a PsPM that can be inverted with nonlinear methods (Bach, Daunizeau, Kuelzow, Friston, & Dolan, 2011). This inversion is necessarily slow. In this paper, we provide a fast approximation to the true solution by using a matching pursuit algorithm (Mallat & Zhang, 1993). This machine learning algorithm is originally designed for fast data compression (for an example in the context of skin conductance, see Chaspari, Tsiartas, Stein, Sermak, & Narayanan, 2015) and finds, by greedy search, a decomposition of the data into atoms from an overcomplete dictionary. We harness this property for model inversion by creating a dictionary of all possible SF that would be allowed under our PsPM, and decomposing the data into most likely SF that constitute the data. Because negative SF are biophysically implausible, we only consider positive weights. Also, the MP algorithm might not return precise SF amplitude estimates, because it considers locally optimal solutions rather than the global set of SF. However, amplitude estimates are crucially required for counting above‐threshold SF (Boucsein et al., 2012). Hence, after identification of the most likely SF, given the data, the algorithm uses multiple regression to reestimate amplitudes of identified SF.

Simulations demonstrate that the algorithm is suboptimal, due to its design. In particular, when the true number of SF is higher than 10 per minute, their number is underestimated. Also, when SF are close together in time (below 4 s), the estimation of their number is less precise. Further, the precision of amplitude and onset estimates linearly decreases with the number of true SF, in contrast to our previous DCM algorithm.

Yet, when analyzing empirical data, both algorithms show similar predictive validity. That is, their ability to separate two different tSA states is comparable. This might imply that the better precision of the DCM inversion yields no empirical benefit. As a possible reason, the average number of SF per minute of data was below 10 in all conditions of all datasets, and hence in a range where MP performed well on simulated data. Further, the model itself is necessarily an approximation to the true psychophysiological relation, and this imprecision might be more severe than the imprecision in the MP model inversion. However, we note that, under some experimental circumstances, the number of SF may exceed 10 per minute. In the experimental paradigms assessed in this paper, tonic arousal was quantified in the absence of external events. In situations with additional external stimulation, or in which participants engage in activities such as producing speech, we anticipate SF to possibly occur with higher frequencies than reported here. In this case, we would not recommend the use of the MP algorithm.

At the same time, computation time for the MP algorithm was 2–3 orders of magnitude faster than for the DCM inversion. While this is a general benefit, it may be of particular relevance for the online quantification of tonic arousal. One important application is biofeedback, where tonic arousal is quantified and fed back to the participant online. Arousal‐driven intervention is another application, for example in advanced driving assistance systems and driver drowsiness detection.

We compared this analysis with visual scoring. Visual scoring had no consistent advantage or disadvantage in terms of predictive validity. At the optimal threshold, it performed significantly better than DCM in one experiment and significantly worse in another. On the other hand, it required more time from the scoring expert than the computation time required by the other methods.

In summary, analysis of empirical data shows that MP is a fast and precise alternative to DCM with comparable accuracy for experimental data. Simulations demonstrate this in particular when the expected number of SF is below 10 per minute and they are separated in time by more than 5 s.

Finally, we observed that, for both methods, the optimal amplitude threshold for the two datasets was different. This was also observed in visual scoring results. Whether this is normal variation between samples, due to the different sample characteristics (males in Dataset 1, females in Dataset 2, mixed in Dataset 3) or the different contrasts tested, cannot be separated in this work and will be a topic of future investigation.
